# Infantile onset carnitine palmitoyltransferase 2 deficiency: Cortical polymicrogyria, schizencephaly, and gray matter heterotopias in an adolescent with normal development

**DOI:** 10.1002/jmd2.12243

**Published:** 2021-09-29

**Authors:** Ivan Shelihan, Elsa Rossignol, Jean‐Claude Décarie, Jean‐Paul Bonnefont, Michèle Brivet, Catherine Brunel‐Guitton, Grant A. Mitchell

**Affiliations:** ^1^ Divisions of Medical Genetics (IS, CBG, GM) and Neurology (ER), Department of Pediatrics CHU Sainte‐Justine and Université de Montréal Montreal Quebec Canada; ^2^ Department of Neurosciences CHU Sainte‐Justine and Université de Montréal Montreal, QC Quebec Canada; ^3^ Department of Medical Imaging CHU Sainte‐Justine and Université de Montréal Montreal Quebec Canada; ^4^ Medical Genetics Federation Necker Enfants Malades Hospital and IMAGINE Institute Paris France; ^5^ Biochemical Diseases, Department of Pediatrics, Faculty of Medicine University of British Columbia, BC Children's Hospital Vancouver British Columbia

**Keywords:** CPT2, carnitine, cerebral, heterotopias, infantile, malformation, palmitoyltransferase, polymicrogyria

## Abstract

**Objective:**

To report an adolescent with infantile‐onset carnitine palmitoyltransferase 2 (CPT2) deficiency and cerebral malformations and to review the occurrence of brain malformations in CPT2 deficiency. The patient presented clinically at age 5 months with dehydration and hepatomegaly. He also has an unrelated condition, X‐linked nephrogenic diabetes insipidus. He had recurrent rhabdomyolysis but normal psychomotor development. At age 17 years, he developed spontaneous focal seizures. Cerebral magnetic resonance imaging revealed extensive left temporo‐parieto‐occipital polymicrogyria, white matter heterotopias, and schizencephaly. Neuronal migration defects were previously reported in lethal neonatal CPT2 deficiency but not in later‐onset forms.

**Design and Methods:**

We searched PubMed, Google Scholar, and the bibliographies of the articles found by these searches, for cerebral malformations in CPT2 deficiency. All antenatal, neonatal, infantile, and adult‐onset cases were included. Exclusion criteria included insufficient information about age of clinical onset and lack of confirmation of CPT2 deficiency by enzymatic assay or genetic testing. For each report, we noted the presence of cerebral malformations on brain imaging or pathological examination.

**Results:**

Of 26 neonatal‐onset CPT2‐deficient patients who met the inclusion criteria, brain malformations were reported in 16 (61.5%). In 19 infantile‐onset cases, brain malformations were not reported, but only 3 of the 19 reports (15.8%) include brain imaging or neuropathology data. In 276 adult‐onset cases, no brain malformations were reported.

**Conclusion:**

To the best of our knowledge, this is the first report of cerebral malformations in an infantile onset CPT2‐deficient patient. Brain imaging should be considered in patients with CPTII deficiency and neurological manifestations, even in those with later clinical onset.

**Abbreviations**
CPT: carnitine palmitoyltransferase (EC 2.3.1.21)
CPT2: carnitine palmitoyltransferase 2

## 1 INTRODUCTION

The hereditary deficiency of carnitine palmitoyltransferase 2 (CPT2, OMIM 600650) is an autosomal recessive inborn error of carnitine and long‐chain fatty acid metabolism.^1^, ^2^, ^3^, ^4^ Three clinical subtypes have been reported: a *lethal neonatal form* (antenatal, OMIM #608836), an *early‐onset infantile* form (hepatic, hepatocardiomuscular, with hypoketotic hypoglycemia, OMIM #600649), and a *late‐onset* (adult) form (myopathic, OMIM #255110).

The *lethal neonatal/antenatal subtype* of CPT2 deficiency is the most severe form. Classically, it presents with acute metabolic signs in the first days of life, accompanied by respiratory distress, hypoglycemia, seizures, hepatomegaly, cardiomegaly, arrhythmias and abnormalities of cardiac conduction, and often mild facial dysmorphic features, cystic renal dysplasia, and brain malformations, including neuronal migration defects (pachygyria), defects of cortical organization (polymicrogyria), brain calcifications or cystic dysplasia, and posterior fossa anomalies (Dandy‐Walker malformations, vermis hypoplasia), often with co‐existing hydrocephalus.^5^, ^6^ Most patients with this subtype die shortly after birth.^5^

*Infantile CPT2 deficiency* shares the metabolic features of the neonatal form,^7^ but signs typically appear between 6 months and 2 years of age, are milder than in the neonatal form and are not known to be accompanied by congenital malformations.^5^, ^6^, ^7^, ^8^

*Late‐onset* (adult) CPT2 deficiency is the commonest form and usually the least severe. It is characterized by episodes of rhabdomyolysis precipitated by prolonged exercise, fasting, high fat intake, exposure to cold, infections, fever, emotional stress and drugs such as ibuprofen, diazepam, and valproic acid.^5^

To date about 30 patients with the *infantile* form and about 300 patients with the *adult* form of CPT2 deficiency have been described.

We report a CPT2‐deficient patient with an infantile age of clinical onset in whom an extensive cortical malformation was discovered following a focal seizure at 17 years of age.

## 2 MATERIALS AND METHODS

We reviewed the patient's medical records from birth until 17 years of age. To test whether other patients with post‐neonatal clinical onset of CPT2 deficiency and cerebral malformations had been reported, we searched PubMed using keywords, as follows: (CPT2 OR “CPT 2” OR CPTII OR “CPT II” OR “Carnitine palmitoyltransferase 2” OR “Carnitine palmitoyltransferase II”) AND (case OR antenatal OR neonatal OR infantile OR hepatocardiomuscular OR hepatic OR “hypoketotic hypoglycemia” OR muscular OR adult OR brain OR cerebral OR neurological OR malformation OR heterotopias OR “migration defect”). We also traced the references cited in the articles found by these searches as necessary.

Exclusion criteria for case histories of neonatal and infantile forms included insufficient information about age of clinical onset and lack of confirmation of CPT2 deficiency by enzymatic assay or genetic testing.

For adult‐onset patients, we did not apply exclusion criteria. The majority of these patients are described in review articles of genotype‐phenotype correlations. Often, only limited clinical data are available. It is not possible to determine from these publications whether some patients are described in more than one study.

Each phenotypic description was analyzed for the presence or absence of documented cerebral malformations by imaging or neuropathology.

## 3 CASE REPORT

### 3.1 Initial presentation

The patient is the first child of healthy, unrelated individuals of Italian descent. The pregnancy was uneventful. He was born at term by spontaneous vaginal delivery. Pediatric visits during the first 5 months showed normal growth and development for his age. The family history revealed no other instances of epilepsy, brain malformations, or metabolic diseases. At 5 months of age, he was admitted because of 2 days of recurrent vomiting and restlessness, with dehydration and a liver edge palpable 4 cm below the costal margin.

Laboratory studies revealed normal blood glucose and lactate and mild‐to‐moderate ketonuria. Plasma aspartate aminotransferase was 500 U/L (reference range, 5‐70 U/L); alanine aminotransferase, 341 U/L (5‐25 U/L); total bilirubin, 27 μmol/L (0‐14 μmol/L); direct bilirubin, 14 μmol/L (0‐4 μmol/L); gamma glutamyl transferase (GGT), 96 U/L (3‐43 U/L); serum sodium 150 mmol/L (130‐142 mmol/L). Urine density was persistently below 1.005. Creatine kinase was not measured.

Abdominal ultrasound revealed hepatomegaly with an echotexture suggesting fatty infiltration. The kidneys appeared normal. Severe hepatic steatosis with hepatomegaly was confirmed on abdominal CT‐scan. Hepatic biopsy showed isolated macrovesicular steatosis. Cardiac ultrasound demonstrated mild septal and left ventricular hypertrophy with normal myocardial function.

Urine organic acids showed acetoacetic acid, 340 μmol/mmol creat. (upper reference level for age, 19 μmol/mmol creat.) and 3‐hydroxybutyric acid, 66 μmol/mmol creat. (upper reference level for age, 36 μmol/mmol creat.) and were otherwise normal.

### 3.2 Diagnostic investigations

The first determination of plasma free carnitine level was normal, with elevated esterified carnitine, 58.4 μmol/L (reference range, 6.4‐31.6 μmol/L). Plasma acylcarnitines showed levels of C16 and C18 species more than 10‐fold above the upper reference value, with lesser elevations of C12, C14 and C2 acylcarnitines.

Fatty acid oxidation studies in cultured fibroblasts showed low oxidation of palmitate and myristate (7.2% and 6.7% of control values, respectively). CPT2 assay in fibroblasts showed CPT2 activity <2.7% of control activity (<0.01 nmol palmitoylcarnitine formed/min/mg protein vs 0.36 in the control).

Sequencing of all *CPT2* exons and surrounding intronic regions revealed compound heterozygosity for two previously reported pathogenic variants, c.887G > A (p.Arg296Gln)^9^ of paternal origin and c.1891C > T (p.Arg631Cys)^10^ of maternal origin (reference sequence, NM_000098).

### 3.3 Subsequent clinical course

Hypernatremia and low urine density persisted. He was found to have a second condition, X‐linked nephrogenic diabetes insipidus, caused by a de novo known pathogenic variant in *AVPR2* (c.541C > T in NM_000054 (p.Arg181Cys).^11^, ^12^, ^13^

He was initially treated by glucose‐containing intravenous fluids then transitioned to frequent nasogastric tube feeding with a low fat, high carbohydrate formula. Plasma aminotransferase levels normalized within days. He had severe gastroesophageal reflux. The treatments of CPT2 deficiency and of nephrogenic diabetes insipidus both require frequent feeds and/or hydration, and therefore gastrostomy was performed at 7 months of age. He received nocturnal gavages until 10 years of age. Subsequently, the gastrostomy was closed and oral hydration at night was prescribed.

After his first hospitalization, intake of long‐chain fatty acids was restricted and he received supplements of medium chain fatty acids and l‐carnitine. Serial abdominal and cardiac ultrasounds showed complete normalization of hepatic morphology at age of 3 years and persistently normal heart function after 13 months of age. He was hospitalized 12 times between 5 and 17 years of age for episodes of rhabdomyolysis precipitated by fever, fasting or intense exercise. He recovered quickly and completely from each episode. Developmental milestones were normal. He had average performance in regular school. A generalized anxiety disorder appeared during adolescence.

**FIGURE 1:**
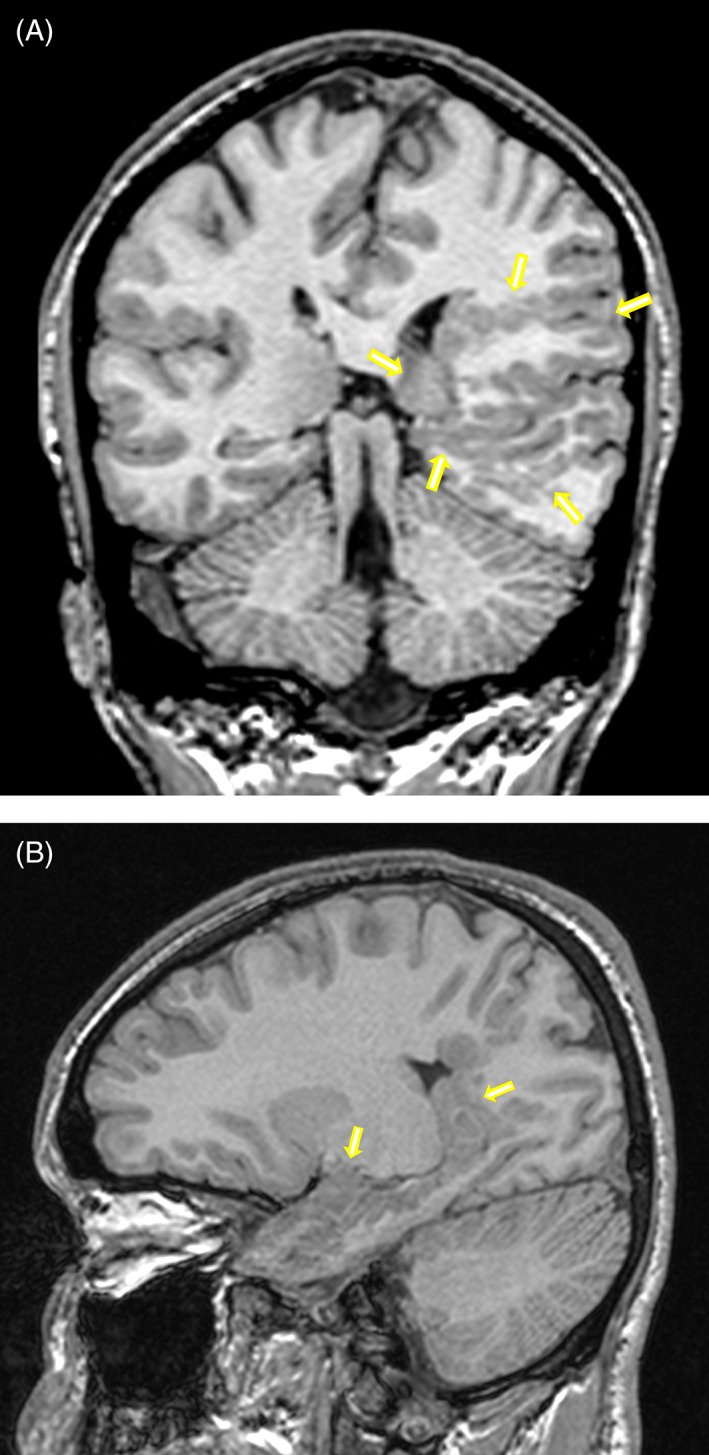
Cerebral magnetic resonance imaging at age 17 years in the carnitine palmitoyltransferase 2 (CPT2)‐deficient patient. Coronal,A, and sagittal, B, 1 mm reformat from a 3D T1 volume acquisition. A, Extensive polymicrogyria, closed lip schizencephaly, and heteropia. B, Extensive heterotopia and abnormal hippocampal formation. The yellow arrows indicate the edge of the malformation

At age 17 years, he experienced two brief focal seizures with secondary generalization (he was found unconscious, with leftward deviation of his head and body, and generalized tonic‐clonic movements). Right‐sided hyperreflexia was present. Brain magnetic resonance imaging (MRI) revealed extensive left parietal, temporal, and occipital polymicrogyria with closed lip schizencephaly, subcortical grey matter heterotopias and a dysplastic left hippocampus (Figure 1A,B). Three electroencephalographic studies performed during follow‐up were normal. He was treated with carbamazepine and remains seizure‐free after 27 months of follow‐up.

### 3.4 Literature review

#### 3.4.1 Neonatal onset patients

Twenty six of 27 reports of neonatal (or antenatal) CPT2 deficiency met the inclusion criteria (Table S1). Sixteen patients (16/26, 61.5%) were reported to have brain malformations. Five of these (31.25%) were consistent with a defect of neuronal migration or cortical organization. Other brain anomalies included: hydrocephalus in four patients (25%); Dandy‐Walker malformation in four (25%); cerebral cystic dysplasia and dysplastic corpus callosum in two patients (12.5%); corpus callosum agenesis and fetal cerebral dysgenesis in two patients (12.5%); brain calcifications in two (12.5%). Some had more than one malformation. In the 10 patients in whom brain malformations were not reported, seven (70%) did not have brain imaging or neuropathology, and therefore a brain malformation cannot be excluded in these individuals (Table 1).

**TABLE 1:** Reported cerebral malformations in CPT2 deficiency

Form	Neonatal/Antenatal	Infantile (including this report)	Adult
Total patients reported	27	26 (27)	276
Patients accepted for further analysis (see Methods)	26	19 (20)	276
Brain imaging or autopsy reported	19	3 (4)	0
Cerebral malformation(s) observed	16^a^	0 (1)	0

^a^ Most frequent anomalies: Defects of neuronal migration or cortical organization (polymicrogyria, heterotopias), 5 (31.25%); Dandy‐Walker malformation, 4 (25%); hydrocephalus, 4 (25%); corpus callosum agenesis or dysplasia, 3 (18.75%); ventriculomegaly, 3 (18.75%); brain calcifications, 2 (12.5%). Some patients had more than one malformation (for details, refer to Table S1).

#### 3.4.2 Infantile onset patients

Twenty‐six infantile‐onset patients were identified in the literature (Table S2). For seven patients (27%), the age of clinical onset was not recorded (Ref. 7 six cases; Ref. 14,one case) and they were excluded. Of note, in these patients, brain malformations were not reported. In the 19 remaining documented infantile CPT2‐deficient patients, brain malformations were not reported. Importantly, only 3 of the 19 (15.8%) of the case reports mentioned brain imaging or neuropathology (Table 1).

#### 3.4.3 Adult onset patients

The search identified 276 patients with the adult muscular form of CPT2 deficiency (Table S3). Brain malformations were not reported in these publications (Table 1).

## 4 DISCUSSION

The clinical spectrum of CPT2 deficiency ranges from congenital malformations with early death to isolated intermittent skeletal muscle involvement in adults. Here, we confirm that cerebral malformations are common (62%) in the neonatal (antenatal) form of CPT2 deficiency and can be present in patients with later clinical presentation.

To the best of our knowledge, this is the first report of a cortical malformation in a CPT2‐deficient patient with clinical onset after the neonatal period. Ohtani et al^15^ described a 17‐month‐old boy with infantile spasms, global developmental delay, and recurrent episodes of rhabdomyolysis. Cerebral MRI revealed bilateral T2‐hyperintense regions in the frontal white matter but no malformations.

Remarkably, the patient remained neurologically asymptomatic for 17 years despite having a large and complex malformation including extensive polymicrogyria, closed lip schizencephaly, subcortical grey matter heterotopias and hippocampal dysplasia. Among 101 patients with malformations of cortical development, aged 1 month to 19 years,^16^ 68% had mental retardation, 73.3% had abnormal neurological examination and 71.3% had epilepsy, with seizure onset typically starting in young children. Their presentation and clinical course were related to the extent and location of the malformation.^16^ By definition, the incidence of asymptomatic brain malformations cannot be determined from such case series: such patients will be underrepresented because imaging studies are usually performed only if neurological signs appear. Clinical experience, however, suggests that such observations are exceptional. Of note, the normal psychomotor development of our patient despite his extensive brain malformations, suggests that the involved regions retained considerable functional capacity.

Both mutations of our patient have previously been reported. c.887G > A (p.Arg296Gln) was reported in trans with a complex allele that produces an in‐frame deletion.^9^ This patient had severe neonatal onset CPT2 deficiency, hydrocephalus, polymicrogyria, and hypoplasia of the cerebellar vermis. The other mutation, c.1891C > T (p.Arg631Cys), has been described in five patients, in a homozygote with clinically severe, infantile‐onset CPT2 deficiency^10^ and also in four homozygotes with isolated muscular signs.^17^ The latter four patients also had two other homozygous variants in *CPT2*, c.1102G > A (p.Val368Ile) and c.1939A > G (p.Met647Val).

**FIGURE 2:**
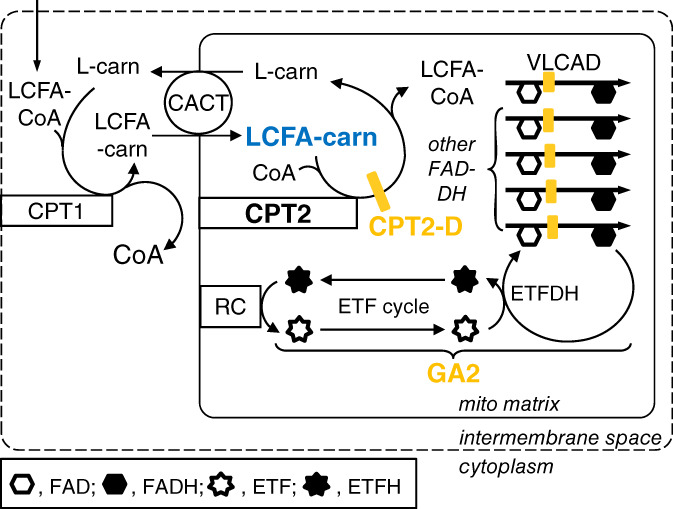
Metabolic proximity of carnitine palmitoyltransferase 2 (CPT2) deficiency (CPT2‐D) and glutaric aciduria type 2 (GA2, OMIM 231680), inborn errors with similar malformations. Long‐chain fatty acids (LCFA) are esterified to LCFA‐CoA in the cytoplasm (not shown), then shuttled into mitochondria. First, LCFA‐carnitines are synthesized near the outer mitochondrial membrane (dotted rectangle) by CPT1. Transport of the LCFA‐carnitine into the mitochondrial matrix is mediated by carnitine‐acylcarnitine translocase (CACT). CPT2 catalyzes reesterification to coenzyme A, forming a LCFA‐CoA for oxidation by very long‐chain acyl‐CoA dehydrogenase (VLCAD). GA2 results either from deficiency of electron transfer flavoprotein (ETF) or of ETF dehydrogenase (ETFDH). GA2 causes the deficiency of multiple flavin‐bound acyl‐CoA dehydrogenases (FAD‐DH) including VLCAD. These enzymes covalently bind flavin adenine dinucleotide (FAD). During catalysis, FAD is reduced to FADH, which must be oxidized in order to restore enzymatic activity. This oxidation is carried out by ETFDH and is coupled to the reduction of ETF to ETFH. ETFH moves to donate an electron to coenzyme Q in the respiratory chain (RC), providing an important fraction of cellular energy supply. Neuronal migration defects and renal cysts can occur both in CPT2 deficiency and in GA2. Both conditions increase LCFA‐carnitines in the mitochondrial matrix, with corresponding increases of plasma LCFA‐carnitines. The mechanistic link, if any, between these metabolic abnormalities and brain and kidney malformations is currently unknown

The mechanisms underlying the occurrence of cerebral malformations in CPT2 deficiency are unknown. Patients may also have renal cysts.^5^, ^6^ Intriguingly, similar brain and kidney malformations also occur in glutaric aciduria type 2, an inborn error affecting multiple flavin‐dependent dehydrogenases, including those of mitochondrial fatty acid oxidation^18^ (Figure 2), but such malformations are not to our knowledge reported in other disorders of fatty acid oxidation, including deficiency of very long‐chain acyl‐CoA dehydrogenase (Figure 2).

The classification of CPT2 deficiency based on age of clinical onset is convenient and often useful, but as this patient shows, the age of clinical onset does not define biologically distinct categories. The patient presented as an infant, evolved to have a classical “adult” course dominated by recurrent episodes of rhabdomyolysis, yet in late adolescence, was found to have a cerebral malformation of prenatal onset.

In practice, clinical descriptions of infantile‐onset CPT2 deficiency are few and are often incomplete. It is not possible to conclude upon the incidence of brain malformations in this form of CPT2 deficiency, but this patient shows that they can occur. Presumably, in most patients, brain malformations cause detectable neurological signs if present, and the decision to perform cerebral imaging depends upon clinical indications. Conversely, the observation of a complex, extensive cerebral malformation that was clinically asymptomatic until adolescence, suggests that cerebral malformation should be considered in any CPT2‐deficient patient who develops unexplained seizures or other neurological signs, regardless of the age of clinical onset.

## CONFLICT OF INTEREST

Ivan Shelihan, Elsa Rossignol, Jean‐Claude Décarie, Jean‐Paul Bonnefont, Michèle Brivet, Catherine Brunel‐Guitton, and Grant A. Mitchell declare that they have no conflict of interest.

## ETHICS APPROVAL

Ethics approval was not required for all research studies.

## PATIENT CONSENT

The patient and his parents provided informed consent for publication of this case report. This article does not contain any studies with human or animal subjects performed by the any of the authors.

## AUTHORS CONTRIBUTIONS

Ivan Shelihan: Data analysis and interpretation and planning and drafting most of the article. Elsa Rossignol: Article contribution and revision. Jean‐Claude Décarie: Article contribution and revision. Jean‐Paul Bonnefont: Article contribution and revision. Michèle Brivet: Article contribution and revision. Catherine Brunel‐Guitton: Article contribution and revision. Grant A. Mitchell: Data analysis and interpretation and planning and drafting of article.

5

References 19‐53 are cited only in Supporting Information, where they are numbered according to the accompanying list, “**References for supplementary tables**”

## Supporting information


**Table S1‐S3** Reports of neonatal (antenatal) form of CPT2 deficiency
