# Supplementary material for: Infantile onset carnitine palmitoyltransferase 2 deficiency: Cortical polymicrogyria, schizencephaly, and gray matter heterotopias in an adolescent with normal development
Source: JIMD Rep. 2021 Sep 29;63(1):3–10. doi: 10.1002/jmd2.12243 (PMC8743346; doi:10.1002/jmd2.12243)
Supplement: Supplementary file 1 — Table S1‐S3 Reports of neonatal (antenatal) form of CPT2 deficiency [file JMD2-63-3-s001.pdf]

Table S1. Relevant reports of the neonatal (antenatal) form of CPT2 deficiency.

Case	Published	Age of presentation	CPT2 activity	Genetic changes in CPT2	Brain imaging/ autopsy data	PMG	HT	CCD	CCA	VM	BC	HC	DWM	PC	FCD	Other	NR
1	Hug et al (1989) [1]	2d	1-17%	NR	No												+
2	Taroni F et al (1994) [2]	At birth	<15%	c.680C>T(p.Pro227Leu)/ c.680C>T(p.Pro227Leu)	Yes	+											
3	North et al (1995) [3]	4d	0-2-22%	NR	Yes		+										
4	Land et al (1995) [4]	At birth	UD	NR	Yes												+
5*	Pierce et al (1999) [5]	1d	NR	NR	Yes			+									
6	Elpeleg et al (2001) [6]	Antenatal	UD	c.1237_1238del(p.Gln413Glufs*8);c.1342T>C(p.Phe448Leu)/ c.1237_1238del(p.Gln413Glufs*8);c.1342T>C(p.Phe448Leu)	Yes				+	+	+						
7	Elpeleg et al (2001) [6]	Antenatal	UD	c.1237_1238del(p.Gln413Glufs*8);c.1342T>C(p.Phe448Leu)/ c.1237_1238del(p.Gln413Glufs*8);c.1342T>C(p.Phe448Leu)	Yes						+						
8	Albers et al (2001) [7]	17h	6-18%	NR	Yes				+								
9	Vladutiu et al (2002) [8]	17h	6%	c.1237_1238del(p.Gln413Glufs*8)/c.112_114delinsGCAGC	No												+
10	Vekemans et al 2003 [9]	4h	<1%	c.983A>G (p.Asp328Gly)/c.983A>G (p.Asp328Gly)	No												+
11	Vekemans et al 2003 [9]	At birth	<1%	c.983A>G (p.Asp328Gly)/c.983A>G (p.Asp328Gly)	No												+
12	Vekemans et al 2003 [9]	At birth	NR	c.983A>G (p.Asp328Gly)/c.983A>G (p.Asp328Gly)	No												+
13	Sigauke et al (2003) [10]	At birth	7%	c.534_558del25insT(p.Leu178_Ile186delinsPhe)/ c.887G>A(p.Arg296Gln)	Yes							+	+				
14	Smeets et al (2003) [11]	1-10d	2%	c.534_558del25insT (p.Leu178_Ile186delinsPhe)/g.5894G>A	No												+
15	Sharma et al (2003) [12]	Died on day 12	7%	NR	Yes												+
16	Isackson et al (2008) [13]	1-2d	9-28%	c.680C>T (p.Pro227Leu)/c.680C>T (p.Pro227Leu)	Yes												+
17	Isackson et al (2008) [13]	1-2d	9-28%	c.1923_1935del(p.Lys642Thrfs)/c.1923_1935del(p.Lys642Thrfs)	Yes	+											
18	Isackson et al (2008) [13]	At birth	28-58%	c.164C>G (p.Pro55Arg)/c.1782delC (Pro595fs)	Yes	+	+							+		+	
19	Isackson et al (2008) [13]	16d	11%	c.1511C>T (p.Pro504Leu)/c.1511C>T (p.Pro504Leu)	No												+
20	Meir et al (2009) [14]	Antenatal	NR	c.1342T>C(p.Phe448Leu)/ c.1342T>C(p.Phe448Leu);c.1237_1238del(p.Gln413Glufs*8)	Yes							+					
21	Meir et al (2009) [14]	Antenatal	NR	c.1342T>C(p.Phe448Leu)/ c.1342T>C(p.Phe448Leu);c.1237_1238del(p.Gln413Glufs*8)	Yes							+					
22	Meir et al (2009) [14]	Antenatal	NR	c.1342T>C(p.Phe448Leu)/ c.1342T>C(p.Phe448Leu);c.1237_1238del(p.Gln413Glufs*8)	Yes	+											
23	Hissink-Muller et al (2009) [15]	Antenatal	NR	c.680C>T (p.Pro227Leu)/c.680C>T (p.Pro227Leu)	Yes				+								
24	Yahyaoui et al (2011) [16]	12d	NR	c.534_558del25insT(p.Leu178_Ile186delinsPhe)/ c.534_558del25insT(p.Leu178_Ile186delinsPhe)	Yes							+	+				
25	Boemer et al (2016) [17]	At birth	NR	c.680C>T (p.Pro227Leu)/c.680C>T (p.Pro227Leu)	Yes					+			+				
26	Boemer et al (2016) [17]	Antenatal	NR	c.680C>T (p.Pro227Leu)/c.680C>T (p.Pro227Leu)	Yes										+		
27	Boemer et al (2016) [17]	At birth	28%	c.680C>T (p.Pro227Leu)/c.680C>T (p.Pro227Leu)	Yes					+			+				

*Excluded because of insufficient documentation (see Methods). NR, Not Reported; UD, Undetectable; PMG, polymicrogyria; HT, heterotopias; CCD, cerebral cystic dysplasia; CCA, corpus callosum anomaly (agenesis or dysplasia); VM, ventriculomegaly; BC, brain calcifications; HC, Hydrocephalus; DWM, Dandy-Walker malformation; PC, periventricular cysts; FCD, fetal cerebral dysgenesis . For more detailed description of cerebral anomalies please refer to source article

Table S2. Relevant reports of the infantile form of CPT2 deficiency

Case	Published	Age of presentation	CPT2 activity (% normal)	Genetic changes in CPT2	Brain imaging/ autopsy data	Structural brain anomalies
1	Demaugre et al (1991) [18]	3m	11.0%	NR	No	NR
2	Taroni et al (1992) [19]	23m	6.6-16.4%	c.1891C>T (p.Arg631Cys)/c.1891C>T (p.Arg631Cys)	No	NR
3	Ohtani et al (1994) [20]	5m	37.0%	Frequent mutations not found	Brain MRI	NR
4	Vianey-Saban et al (1995) [21]	40d	<15%	NR	No	NR
5	Yamamoto et al (1996) [22]	6m	3.0%	c.1148T>A (p.Phe383Tyr)/c.1931T>C (p.Leu644Ser)	No	NR
6	Yamamoto et al (1996) [22]	9m	3.0%	c.520G>A (p.Glu174Lys)/c.1148T>A (p.Phe383Tyr)	No	NR
7	Wataya et al (1998) [23]	9m	6.3%	c.1148T>A (p.Phe383Tyr)/c.1148T>A (p.Phe383Tyr)	No	NR
8	Fontaine et al (1998) [24]	6w	7.0%	NR	No	NR
9*	Yang et al (1998) [25]	NR	NR	c.1810C>T (p.Pro604Ser)/Unknown	No	NR
10	Hurvitz et al (2000) [26]	8m	16.5%	c.338C>T (p.Ser113Leu)/c.338C>T (p.Ser113Leu)	No	NR
11	Vladutiu et al (2002) [8]	11m	17.0%	c.1237_1238del (p.Gln413Glufs*8/c.149C>A (p.Pro50His)	No	NR
12*	Thuillier et al (2003) [27]	NR	4-12%	c.452G>A (p.Arg151Gln)/c.452G>A (p.Arg151Gln)	No	NR
13*	Thuillier et al (2003) [27]	NR	4-12%	c.983A>G (p.Asp328Gly)/c.983A>G (p.Asp328Gly)	No	NR
14*	Thuillier et al (2003) [27]	NR	4-12%	c.983A>G (p.Asp328Gly)/c.983A>G (p.Asp328Gly)	No	NR
15*	Thuillier et al (2003) [27]	NR	4-12%	c.1148T>A (p.Phe383Tyr)/Unknown	No	NR
16*	Thuillier et al (2003) [27]	NR	4-12%	c.1237_1238del(p.Gln413Glufs*8);c.1342T>C(p.Phe448Leu)/<br>c.1237_1238del(p.Gln413Glufs*8);c.1342T>C(p.Phe448Leu)	No	NR
17*	Thuillier et al (2003) [27]	NR	4-12%	c.1883A>C (p.Tyr628Ser)/c.1883A>C (p.Tyr628Ser)	No	NR
18	Spiegel et al (2007) [28]	3m	3.4-7%	c.1507C>T (p.Arg503Cys)/c.1507C>T (p.Arg503Cys)	No	NR
19	Isackson et al (2008) [13]	15m	2.5%	c.359A>G (p.Tyr120Cys)/c.359A>G (p.Tyr120Cys)	No	NR
20	Yasuno et al (2008) [29]	1m	18.5%	c.1148T>A (p.Phe383Tyr)/c.1813G > C (p.V605L)	No	NR
21	Yasuno et al (2008) [29]	2y	14.4%	c.1148T>A (p.Phe383Tyr)/ Unknown	No	NR
22	Yasuno et al (2008) [29]	6m	5.6%	c.1148T>A (p.Phe383Tyr)/Unknown	No	NR
23	Yasuno et al (2008) [29]	11y?	10.0%	c.1148T>A (p.Phe383Tyr)/Unknown	No	NR
24	Hori et al (2010) [30]	15m	14-22%	c.520G>A (p.Glu174Lys)/Unknown	Brain MRI	NR
25	Bouchireb et al (2010) [31]	10m	NR	c.1883A>C (p.Tyr628Ser)/c.1883A>C (p.Tyr628Ser)	Whole body MRI	NR
26	Yamamotoa et al (2011) [32]	6m	NR	c.1148T>A (p.Phe383Tyr)/c.1931T>C (p.Leu644Ser)	No	NR

*Excluded because of insufficient documentation (see Methods). NR-Not Reported

Table S3. Relevant reports of the late-onset adult form of CPT2 deficiency

**Published**	**Number of cases**	**Structural brain anomalies**
Taggart et al (1999)[33]	13	NR
Wieser et al. (2003)[34]	32	NR
Isackson et al (2006)[35]	101	NR
Corti et al. (2007)[36]	22	NR
Anichini et al (2011)[37]	9*	NR
Fanin et al. (2012)[38]	49	NR
Joshi et al. (2014) [39]	50	NR
**Total**	**276**	

* 16 other patients in this study were removed because they are also described by Fanin et al [39]. NR-Not Reported

References for supplementary tables

[1] Hug G, Soukup S, Berry H, et al. Carnitine palmityl transferase (CPT): deficiency of CPT II but not of CPT I with reduced total and free carnitine but increased acylcarnitine. Pediat Res. 1989;25:115

[2] Taroni F, Gellera C, Cavadini P, et al. Lethal carnitine palmitoyltransferase (CPT) II deficiency in newborns: a molecular-genetic study. AmJ Hum Genet. 1994;55:A265.

[3] North KN, Hoppel CL, De Girolami U, et al. Lethal neonatal deficiency of carnitine palmitoyltransferase II associated with dysgenesis of the brain and kidneys. J Pediatr. 1995;127(3):414-420.

[4] Land JM, Mistry S, Squier M, et al. Neonatal carnitine palmitoyltransferase-2 deficiency: a case presenting with myopathy. Neuromuscul Disord. 1995;5:129-137.

[5] Pierce MR, Pridjian G, Morrison S, Pickoff AS. Fatal carnitine palmitoyltransferase II deficiency in a newborn: new phenotypic features. Clin Pediatr. 1999;38(1):13-20.

[6] Elpeleg ON, Hammerman C, Saada A, et al. Antenatal presentation of carnitine palmitoyltransferase II deficiency. Am J Med Genet. 2001;102(2):183-187.

[7] Albers S, Marsden D, Quackenbush E, et al. Detection of neonatal carnitine palmitoyltransferase II deficiency by expanded newborn screening with tandem mass spectrometry. Pediatrics. 2001;107.

[8] Vladutiu GD, Quackenbush EJ, Hainline BE, Albers S, Smail DS, Bennett MJ. Lethal neonatal and severe late infantile forms of carnitine palmitoyltransferase II deficiency associated with compound heterozygosity for different protein truncation mutations. J Pediatr. 2002;141:734-736.

[9] Vekemans BC, Bonnefont JP, Aupetit J, et al. Prenatal diagnosis of carnitine palmitoyltransferase 2 deficiency in chorionic villi: a novel approach. Prenatal Diag. 2003;23:884-887.

[10] Sigauke E, Rakheja D, Kitson K, Bennett MJ. Carnitine palmitoyltransferase II deficiency: a clinical, biochemical, and molecular review. Lab Invest. 2003;83(11):1543-1554.

[11] Smeets RJ, Smeitink JA, Semmekrot BA, et al. A novel splice site mutation in neonatal carnitine palmitoyl transferase II deficiency. J Hum Genet. 2003;48(1):8-13.

[12] Sharma R, Perszyk AA, Marangi D, Monteiro C, Raja S. Lethal neonatal carnitine palmitoyltransferase II deficiency: an unusual presentation of a rare disorder. Am J Perinatol. 2003;20(1):25-32.

[13] Isackson PJ, Bennett MJ, Lichter-Konecki U, et al. CPT2 gene mutations resulting in lethal neonatal or severe infantile carnitine palmitoyltransferase II deficiency. Mol Gen Metab. 2008;94:422-427.

[14] Meir K, Fellig Y, Meiner V, et al. Severe infantile carnitine palmitoyl-transferase II deficiency in 19-week fetal sibs. Pediatr Dev Pathol. 2009;12(6):481-486.

[15] Hissink-Muller P, Lopriore E, Boelen C, Klumper F, Duran M, Walther F. Neonatal carnitine palmitoyltransferase II deficiency: failure of treatment despite prolonged survival. BMJ Case Rep. 2009;2009:bcr02.2009.1550.

[16] Yahyaoui R, Espinosa MG, Gómez C, et al. Neonatal carnitine palmitoyltransferase II deficiency associated with Dandy-Walker syndrome and sudden death. Mol Genet Metab. 2011;104:414-416.

[17] Boemer F, Deberg M, Schoos R, et al. Diagnostic pitfall in antenatal manifestations of CPT II deficiency. Clin Genet. 2016;89:193-197.

[18] Demaugre F, Bonnefont JP, Colonna M, Cepanec C, Leroux JP, Saudubray JM. Infantile form of carnitine palmitoyltransferase II deficiency with hepatomuscular symptoms and sudden death. Physiopathological approach to carnitine palmitoyltransferase II deficiencies. J Clin Invest. 1991;87(3):859-864.

[19] Taroni F, Verderio E, Fiorucci S, et al. Molecular characterization of inherited carnitine palmitoyltransferase II deficiency. Proc Natl Acad Sci U S A. 1992;89(18):8429-8433.

[20] Ohtani Y, Tomoda A, Miike T, Matsukura M, Miyatake M, Narazaki O. Central nervous system disorders and possible brain type carnitine palmitoyltransferase II deficiency. Brain Dev. 1994;16(2):139-145.

[21] Vianey-Saban C, Stremler N, Paut O, et al. Infantile form of carnitine palmitoyltransferase II deficiency in a girl with rapid fatal onset. J Inher Metab Dis. 1995;18:362-363.

[22] Yamamoto S, Abe H, Kohgo T, et al. Two novel gene mutations (Glu174-->Lys, Phe383-->Tyr) causing the "hepatic" form of carnitine palmitoyltransferase II deficiency. Hum Genet. 1996;98(1):116-118.

[23] Wataya K, Akanuma J, Cavadini P, et al. Two CPT2 mutations in three Japanese patients with carnitine Palmitoyltransferase II deficiency: functional analysis and association with polymorphic haplotypes and two clinical phenotypes. Hum Mutat. 1998;11:377.386.

[24] Fontaine Monique, Briand Gilbert, Largillière Claude, Degand Pierre, Divry Priscille, Vianey-Saban Christine, Mousson Bénédicte, Vamecq Joseph. Metabolic studies in a patient

with severe carnitine palmitoyltransferase type II deficiency. Clinica Chimica Acta. 1998;273 (2):161–170.

[25] Yang BZ, Ding JH, Dewese T, et al. Identification of four novel mutations in patients with carnitine Palmitoyltransferase II (CPT II) deficiency. Mol Genet Metab. 1998;64(4):229-236.

[26] Hurvitz H, Klar A, Korn-Lubetzki I, Wanders RJA, Elpeleg ON. Muscular carnitine palmitoyltransferase II deficiency in infancy. Pediatr Neurol. 2000;22(2):148-150.

[27] Thuillier L, Rostane H, Droin V, et al. Correlation between genotype, metabolic data, and clinical presentation in carnitine palmitoyltransferase 2 (CPT2) deficiency. Hum Mutat. 2003;21:493-501.

[28] Spiegel R, Shaag A, Gutman A, et al. Severe infantile type of carnitine palmitoyltransferase II (CPT II) deficiency due to homozygous R503C mutation. J Inherit Metab Dis. 2007.

[29] Yasuno T, Kaneoka H, Tokuyasu T, et al. Mutations of carnitine palmitoyltransferase II (CPT II) in Japanese patients with CPT II deficiency. Clin Genet. 2008;73(5):496-501.

[30] Hori T, Fukao T, Kobayashi H, et al. Carnitine palmitoyltransferase 2 deficiency: the time-course of blood and urinary acylcarnitine levels during initial L-carnitine supplementation. Tohoku J Exp Med. 2010;221(3):191-195.

[31] Bouchireb K, Teychene A-M, Rigal O, et al. Post-mortem MRI reveals CPT2 deficiency after sudden infant death. Eur J Pediatr. 2010;169:1561-1563.

[32] Yamamoto T, Tanaka H, Kobayashi H, et al. Retrospective review of Japanese sudden unexpected death in infancy: the importance of metabolic autopsy and expanded newborn screening. Mol Genet Metab. 2011;102:399-406.

[33] Taggart RT, Smail D, Apolito C, Vladutiu GD. Novel mutations associated with carnitine palmitoyltransferase II deficiency. Hum Mutat. 1999;13(3):210-220.

[34] Wieser T, Deschauer M, Olek K, Hermann T, Zierz S. Carnitine palmitoyltransferase II deficiency: molecular and biochemical analysis of 32 patients. Neurology. 2003;60(8):1351-1353.

[35] Isackson PJ, Bennett MJ, Vladutiu GD, et al. Identification of 16 new disease-causing mutations in the CPT2 gene resulting in carnitine palmitoyltransferase II deficiency. Mol Genet Metab. 2006;89(4):323-323, 331.

[36] Corti S, Bordoni A, Ronchi D, et al. Clinical features and new molecular findings in carnitine palmitoyltransferase II (CPT II) deficiency. J Neurol Sci. 2008;266(1–2):97-103.

[37] Anichini A, Fanin M, Vianey-Saban C, et al. Genotype-phenotype correlations in a large series of patients with muscle type CPT II deficiency. Neurol Res. 2011;33(1):24-32.

[38] Fanin M, Anichini A, Cassandrini D, et al. Allelic and phenotypic heterogeneity in 49 Italian patients with the muscle form of CPT-II deficiency. Clin Genet. 2012;82:232-239.

[39] Joshi PR, Deschauer M, Zierz S. Clinically symptomatic heterozygous carnitine palmitoyltransferase II (CPT II) deficiency. Wien Klin Wochenschr. 2012;124(23–24):851-854.
